# Video‐assisted thoracoscopic excision of esophageal duplication cyst in an adult

**DOI:** 10.1002/ccr3.2457

**Published:** 2019-09-27

**Authors:** Bassam Darwish, Mohammad Bashar Izzat

**Affiliations:** ^1^ Department of Surgery Faculty of Medicine Damascus University Damascus Syrian Arab Republic

**Keywords:** duplication cyst, esophagus, minimally invasive, surgery, thoracoscopy

## Abstract

Esophageal duplication cysts are rarely encountered in adult patients. Complete cyst resection is achievable employing video‐assisted thoracoscopic surgical techniques, with low morbidity and excellent cosmesis.

## Graphical abstract

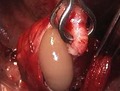

## 1 INTRODUCTION

Esophageal duplication cysts (EDCs) are well‐documented, yet distinctively rare embryonic anomalies of the upper digestive tract.^1^ EDCs most frequently involve the lower esophagus and may possibly trigger a wide array of symptoms, subject to their individual size and location within the posterior mediastinum. Manifestations may also follow the delayed development of certain complications such as cyst infection, bleeding, spontaneous rupture, or fistulation.^2^

Reports of EDCs presenting beyond childhood are scarce in the medical archives.^3^, ^4^, ^5^ The present unique case of an adult patient with a symptomatic EDC is reported so as to underscore the benefits of the minimally invasive surgical approach.

## 2 CASE REPORT

A 35‐year‐old male was hospitalized for a 4 months complaint of worsening difficulty in swallowing, predominantly to solids, accompanied by mild loss of weight. Hematology and biochemistry laboratory tests were within normal limits, and standard chest radiography did not reveal any lung or mediastinal lesions.

**Figure 1:**
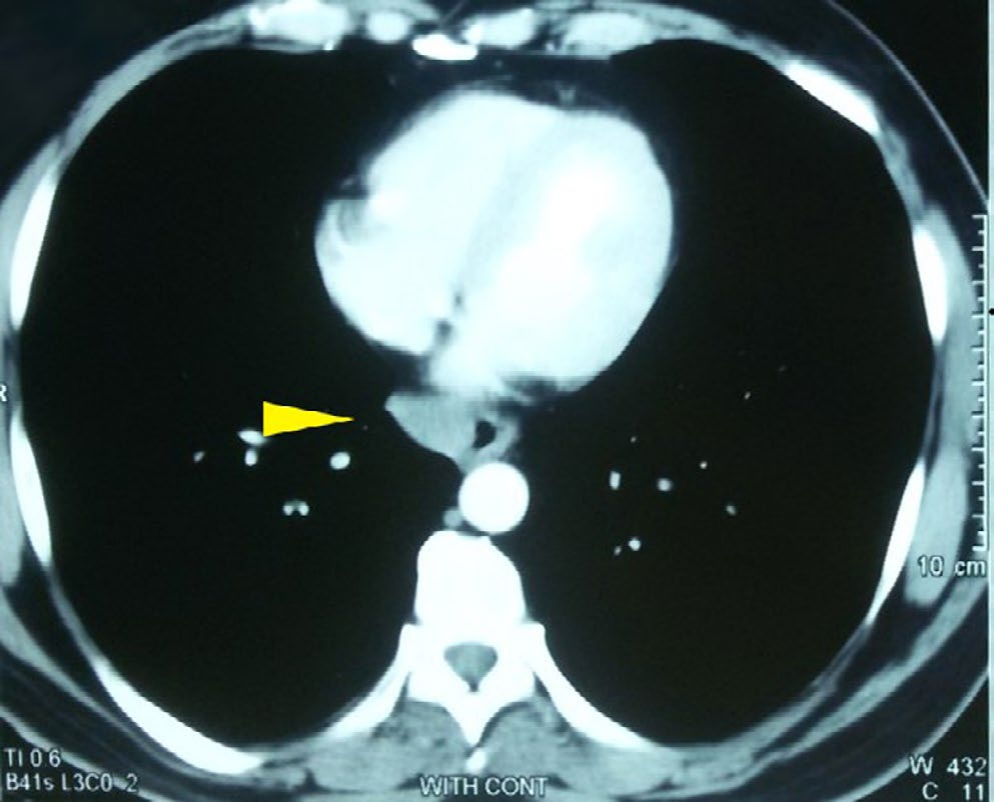
Contrast‐enhanced computed tomography of the chest showing a well‐defined homogeneous mass (yellow arrow head) on the right side of the distal esophagus

Contrast‐enhanced computed tomography of the chest showed a well‐defined 3 × 3.5 × 4 cm homogeneous mass on the right side of the distal esophagus, proximal to the gastro‐esophageal junction (Figure 1). Transesophageal ultrasound was not available, but upper gastrointestinal endoscopy showed a smooth submucosal bulge in the distal segment of the esophagus, resulting in evident localized narrowing of the esophageal lumen. The overlying mucosa was intact.

**Figure 2:**
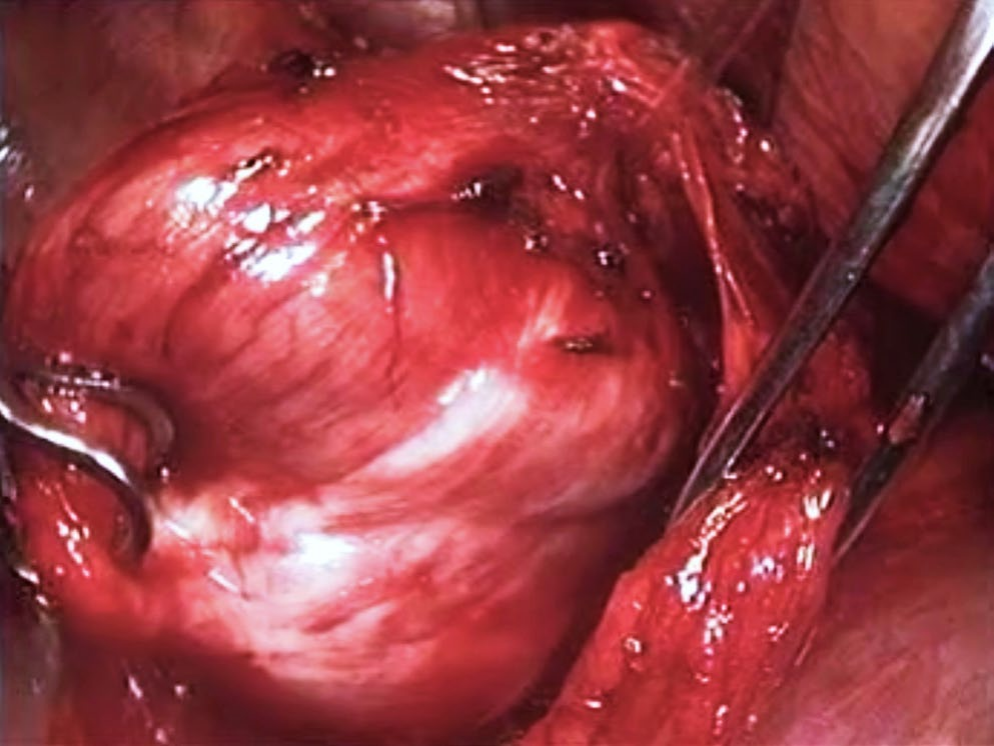
Intraoperative appearance of esophageal duplication cyst, grasped by a clamp

**Figure 3:**
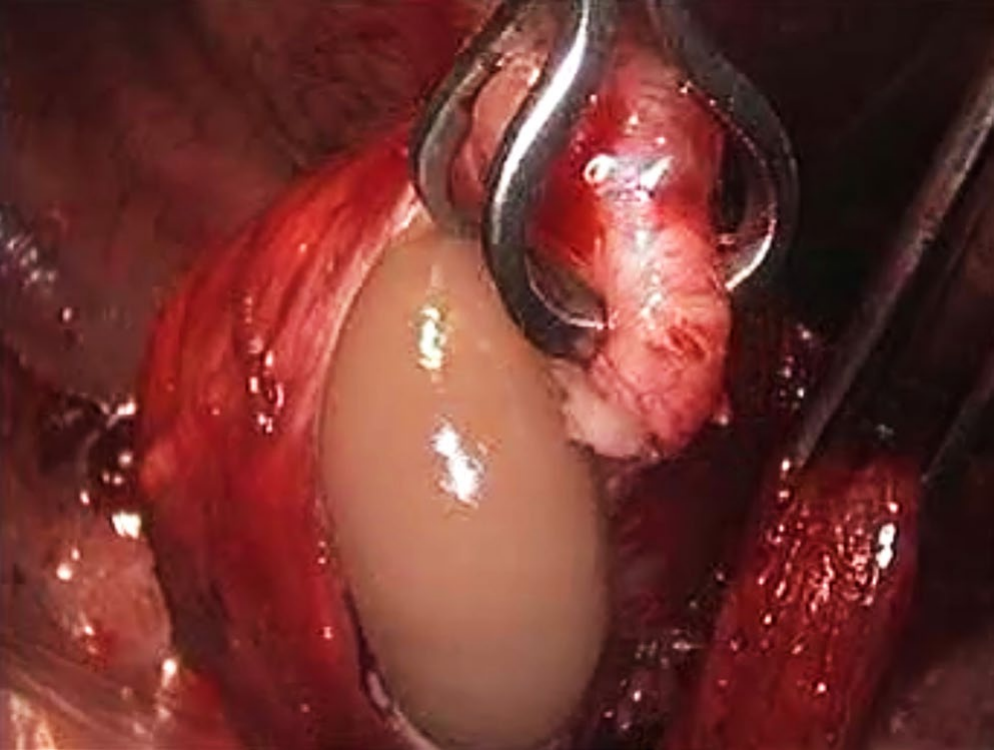
Thick creamy fluid leaking upon incising the lesion

**Figure 4:**
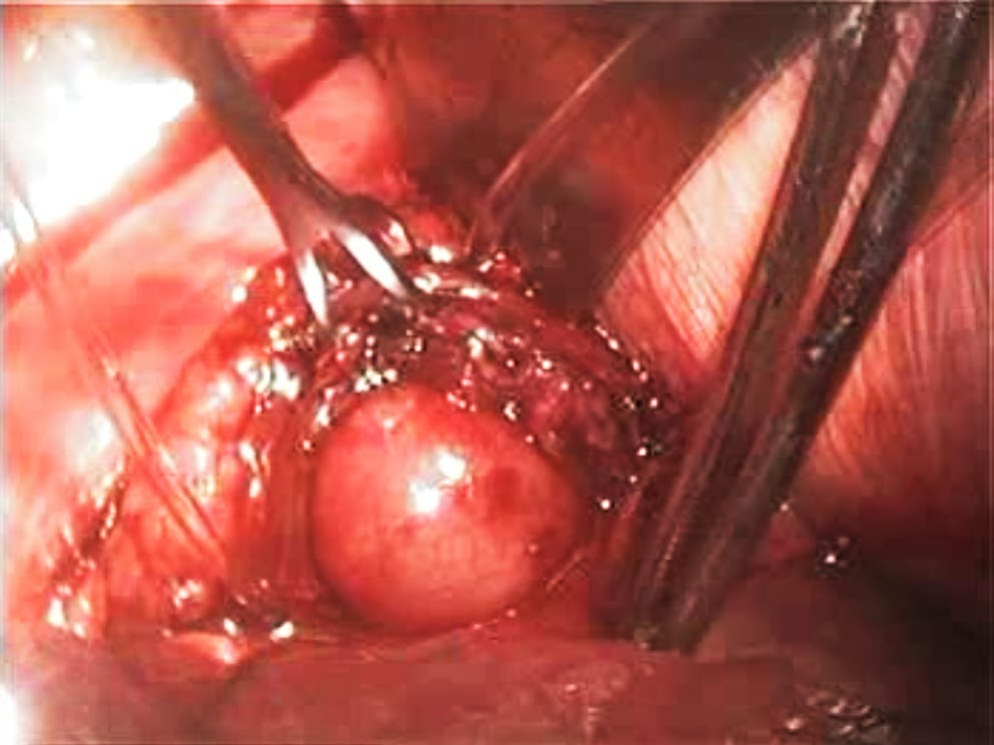
Bulging of the intact esophageal mucosa upon infusing methylene blue solution into the esophageal lumen

Decision was made to perform elective excision of this mass lesion and, since the distal esophagus is usually more accessible from the left, this was carried out employing a left‐sided video‐assisted thoracoscopic surgical (VATS) approach. Using three 5‐mm ports, the lower esophagus was dissected and the mass was identified (Figure 2). Attempts to enucleate the mass were unsuccessful and, upon incising the mass to perform a frozen section procedure, thick creamy fluid leaked out confirming its cystic nature (Figure 3). Consequently, layers of the decompressed cyst were dissected off the esophageal musculature with ease, the cyst was excised, and the specimen was retrieved through a limited thoracotomy incision. The continuity of the esophageal mucosa was confirmed by infusing methylene blue solution into the esophageal lumen while occluding the esophagus at the gastro‐esophageal junction (Figure 4). The muscular edges of the esophagus were reapproximated, and the resection site was buttressed with the mediastinal pleura.

There were no postoperative complications. The patient returned gradually to full oral intake and was discharged home on the fourth postoperative day in an excellent general condition. He remains symptom‐free after 4 months of follow‐up. Histology confirmed the lesion to be an EDC, with dual muscular layer and a characteristic columnar ciliated epithelial lining surrounded by severe chronic inflammatory infiltrate.

## 3 DISCUSSION

Open surgical resection via conventional posterolateral thoracotomy has long been the standard approach to the treatment of EDCs^1^; nevertheless, the benefits of the minimally invasive surgical techniques have already been extended to the management of EDCs.^6^ An increasing number of publications report satisfactory clinical outcomes with VATS resection of EDCs beyond practicality and safety.^6^, ^7^, ^8^ Compared to open surgical resection, the VATS approach has been observed to be associated with shorter periods of chest tube drainage and inhospital stays, and lower rates of intraoperative complications, such as tracheal or esophageal injuries.^6^, ^7^, ^8^

Published experiences to date with the VATS approach to EDCs have predominantly been in managing pediatric patients, whereas reports of VATS resection of EDCs in adults are still limited to a handful of cases only.^7^, ^8^ This is conceivably due to the distinctive scarceness of adult EDCs in clinical practice.^3^, ^4^, ^5^ The present case demonstrates that elective VATS resection of EDCs in adults is safe and can be achieved with minimal morbidity. Furthermore, it highlights additional benefits of the VATS magnification. Specifically, despite the difficult low location of the lesion in our patient, we were able to obtain wide access to the intramural cyst, to dissect its layers off the adjacent esophageal wall safely without perforating the esophageal mucosa, and to preserve both vagus and phrenic nerves.

This report extends contemporary archives on the use of VATS techniques to the removal of intramural EDCs in adult patients. Complete VATS resection of an EDC was both safe and achievable with low morbidity and excellent cosmesis. We propose that this approach may be the new surgical technique of choice.

## CONFLICT OF INTEREST

None declared.

## AUTHOR CONTRIBUTIONS

Bassam Darwish: contributed to conception and acquisition of data, drafted the manuscript and revised it critically, and gave final approval of the version to be published. Mohammad Bashar Izzat: contributed to conception and acquisition of data, drafted the manuscript and revised it critically, and gave final approval of the version to be published.

## ETHICAL APPROVAL

All procedures performed in this study were in accordance with the ethical standards of the Damascus University Research Ethics Committee and with the 1964 Helsinki declaration and its later amendments.

## INFORMED CONSENT

Informed consent was obtained from all individual participants included in the study.
